# The Effects of Intravitreal Bevacizumab in Infectious and Noninfectious Uveitic Macular Edema

**DOI:** 10.1155/2014/729465

**Published:** 2014-07-21

**Authors:** Hassan Al-Dhibi, Issam H. Hamade, Ali Al-Halafi, Maan Barry, Charbel Bou Chacra, Vishali Gupta, Khalid F. Tabbara

**Affiliations:** ^1^King Khaled Eye Specialist Hospital, Al-Oruba Street, P.O. Box 7191, Riyadh 11462, Saudi Arabia; ^2^The Eye Center and the Eye Foundation for Research in Ophthalmology, Riyadh, Saudi Arabia; ^3^Department of Ophthalmology, College of Medicine, King Saud University, Riyadh, Saudi Arabia; ^4^The Wilmer Ophthalmological Institute, The Johns Hopkins University School of Medicine, Baltimore, MD, USA

## Abstract

*Background/Aims.* To assess the effect of intravitreal bevacizumab injection (IVBI) for the treatment of macular edema due to infectious and noninfectious uveitides. *Design.* Retrospective interventional case series.* Methods.* A chart review was performed on all the patients who were diagnosed with uveitic macular edema (UME) and received 1.25 mg of IVBI at two referral centers in Riyadh, Saudi Arabia. All included patients had their visual acuity and macular thickness analyzed at baseline and at 1 and 3 months following IVBI and any sign of reactivation was noted. *Results.* The mean age of patients was 41 ± 16 years with a mean followup of 4 ± 1 months. Ten patients had idiopathic intermediate uveitis, 9 patients had Behcet's disease, 10 had idiopathic panuveitis, and twelve patients had presumed ocular tuberculosis uveitis. Following IVBI, the mean LogMAR visual acuity improved from 0.8 ± 0.8 at baseline to 0.4 ± 0.5 at 1 month and 0.3 ± 0.5 at 3 months (*P* < 0.002, at 3 months). The mean macular thickness was 430 ± 132 *μ*m at baseline. Following IVBI macular thickness improved to 286 ± 93 *μ*m at 1 month and to 265 ± 88 *μ*m at 3 months of followup (*P* < 0.001, at 3 months). *Conclusion.* Bevacizumab was effective in the management of UME associated with both infectious and noninfectious uveitides. Intravitreal bevacizumab induced remission of UME with infectious uveitis and had no immunosuppressive effect against infectious agents.

## 1. Introduction

Uveitic macular edema (UME) occurs in up to 33% of uveitis cases and represents the most common cause of visual loss in patients with uveitis [1, 2]. The underlying pathophysiology of macular edema in uveitis is not well understood. However, several factors may play a role in the development of the edema including inflammatory cytokines, such as interferon gamma, interleukin 2, interleukin 6, interleukin 10, tumor necrosis factor alpha, and vascular endothelial growth factor (VEGF) [3–7].

In patients with uveitis and macular edema, greater concentrations of VEGF are upregulated compared to those without UME. Additionally, VEGF significantly stimulates and increases vascular permeability [7–10].

Early medical treatment is advocated to suppress intraocular inflammation and to prevent progressive and irreversible damage to the macular photoreceptors secondary to chronic and persistent UME [4]. Current management of UME includes the use of topical nonsteroidal anti-inflammatory, oral, periocular, and intraocular injections of corticosteroids as well as oral carbonic anhydrase inhibitors, systemic somatostatin analogs, interferon alpha, mycophenolate mofetil, and VEGF inhibitors [11–20]. However, uveitic macular edema may be nonresponsive to these treatments and continue to progress despite the control of ocular inflammation.

Bevacizumab is a recombinant humanized full-length monoclonal antibody against VEGF that has been used off-label for the treatment of age-related choroidal neovascularization (CNV) and other ocular pathologies that include UME [21–28]. Several clinical reports have described improved visual acuity and a reduction or resolution of macular edema in patients with noninfectious uveitis following intravitreal bevacizumab or ranibizumab injection as an adjunct therapy [10, 29–34]. However, the behavior and response of macular edema due to different etiologies have not been analyzed in detail. The present study aims to compare the effect of intravitreal bevacizumab in uveitic macular edema in patients with different etiologies: idiopathic intermediate uveitis, Behcet's disease, idiopathic panuveitis, and presumed ocular tuberculosis uveitis.

## 2. Patients and Methods

Patient charts were reviewed for cases of uveitic macular edema who had central 1.00 mm macular thickness by OCT of >250 *μ*m and underwent intravitreal bevacizumab injection between June 2006 and June 2009 at King Khaled Eye Specialist Hospital (KKESH) and The Eye Center in Riyadh, Saudi Arabia. Four groups were included in the study: idiopathic intermediate uveitis (IIU), Behcet's disease (BD), idiopathic panuveitis (IPU), and presumed ocular tuberculosis uveitis (POTBU). The intravitreal dosage was 1.25 mg of bevacizumab (Avastin, Genentech/Roche) and repeated as required. Inclusion criteria were patients with refractory UME that was nonresponsive to topical, periocular, or intraocular injections of corticosteroids or different systemic therapy for uveitis within the previous 3 months. Patients with UME associated with epiretinal membrane or vitreomacular traction, pregnant patients, and patients who underwent cataract or intraocular surgeries during the study period were excluded. The study was approved by the IRB.

Demographic data on age and gender of the cohort were collected. The outcome measures included baseline logarithm of the minimal angle of resolution (LogMAR), visual acuity, and macular thickness. Data were collected at 1 and 3 months after intravitreal bevacizumab. The 1 mm central macular thickness was measured with optical coherence tomography (OCT) (Stratus III, Carl Zeiss Meditec, Dublin, CA, USA). The time of onset of macular edema or ocular complications and the follow-up period were recorded. The numbers of intravitreal injections of bevacizumab were recorded. Fluorescein angiography was performed on all patients to record the UME before and after treatment. All topical and systemic medications such as methotrexate, cyclosporine, azathioprine, steroids, infliximab, and antituberculosis therapy were continued during the follow-up period as required.

The diagnosis of presumed ocular tuberculosis was made based on clinical findings of chorioretinitis, granulomatous uveitis, positive PPD of 15 mm of induration or greater, positive response to antituberculosis therapy within 4 weeks, and exclusion of other causes of uveitis as previously reported [35]. Minimum followup was three months. The institutional review boards of both study centers approved this study.

### 2.1. Intravitreal Bevacizumab

After discussing the details of the intravitreal injection with each patient, all patients read and signed an informed consent prior to the procedure. The pupil was dilated, and topical anesthesia and topical moxifloxacin 0.5% were instilled. The lids and lashes were cleansed with povidone iodine 10% solution and a sterile drape was placed over the eye. A sterile lid speculum was inserted. Povidone iodine 5% ophthalmic solution was instilled and, after 90 seconds, rinsed with saline solution. A swab soaked in 5% povidone iodine was placed on the conjunctiva at the site of injection. A 0.05 mL solution containing 1.25 mg of bevacizumab was injected intravitreally. The bevacizumab was prepared in the compounding pharmacy. The injection site was 3.5 mm posterior to the limbus for phakic patients and 3 mm for pseudophakic and aphakic patients and injection was performed with a 30-gauge needle avoiding the horizontal meridians and aiming at the center of the globe. Broad spectrum antimicrobial eye drops were instilled at the end of the procedure and patients were instructed to continue topical antimicrobial drops four times daily for one week. Patients were requested to return at weekly intervals.

### 2.2. Control of Inflammation and Repeated Intravitreal Injections

Intraocular inflammation was graded during each follow-up visit based on the recommendations of the Standardization of Uveitis Nomenclature (SUN) working group [36]. The number of intravitreal injections of bevacizumab was correlated with the activity of the disease. Retreatments of intravitreal bevacizumab (up to one injection per month) were performed as required during the three-month follow-up period. The pre- and postinjection visual acuity was converted from Snellen to LogMAR scale.

### 2.3. Statistical Analyses

Descriptive statistics such as means, standard deviation, and percentages were calculated. Statistical analyses were performed to determine the mean change from baseline visual acuity to 1 month and 3 months of followup. The mean change from baseline retinal thickness using OCT was analyzed at 1 and 3 months. Statistical analyses were performed using repeated measure analyses of variance (ANOVA). All *P* values were two-sided and the significance level was set at 0.05. Data analyses were performed with SPSS for Windows version 11.0 (SPSS Inc., Chicago, IL, USA).

## 3. Results

**Figure 1:**
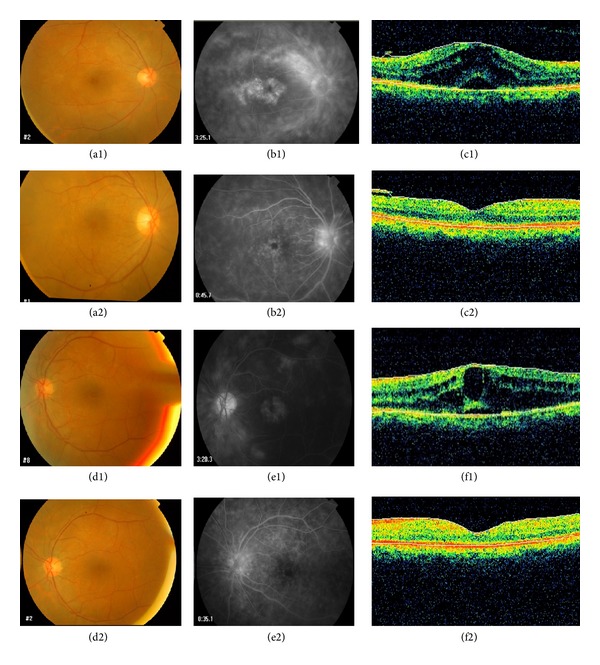
A 56-year-old female with bilateral idiopathic intermediate uveitis and chronic cystoid macular edema. (a1), (b1), and (c1) and (d1), (e1), and (f1) are the fundus photos, fluorescein angiograms, and optical coherence tomography prior to treatment with intravitreal bevacizumab in both eyes. (a2), (b2), and (c2) and (d2), (e2), and (f2) are the fundus photos, fluorescein angiograms and optical coherence tomography, after treatment with intravitreal bevacizumab, which show the response of CME after intravitreal bevacizumab.

**Figure 2:**
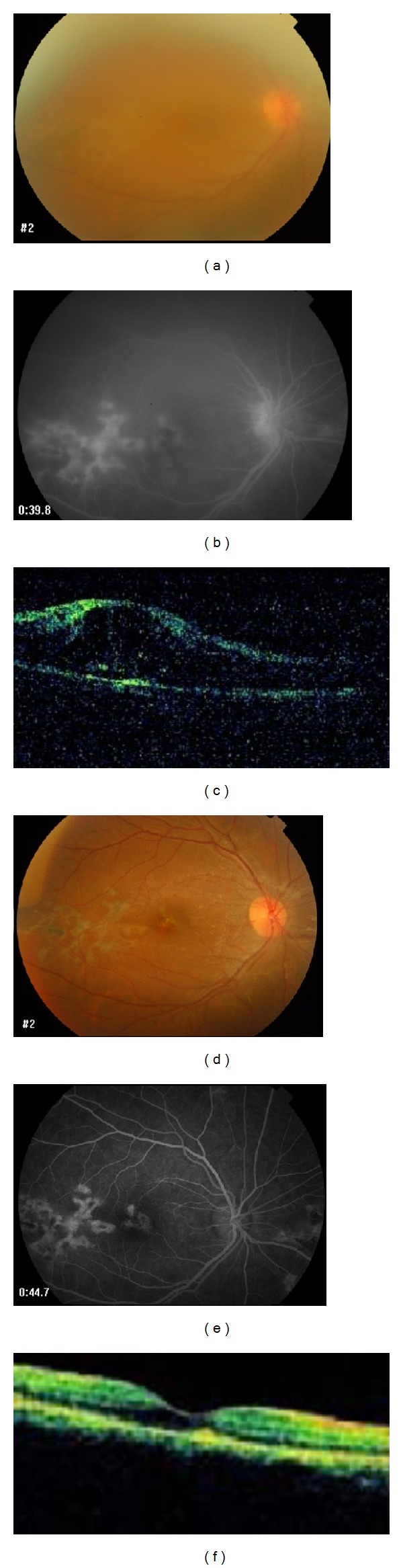
A 28-year-old female with presumed intraocular tuberculosis, choroiditis, and cystoid macular edema in the right eye. (a), (b), and (c) are the fundus photos, fluorescein angiograms, and optical coherence tomographies, prior to treatment with intravitreal bevacizumab. (d), (e), and (f) are the fundus photos, fluorescein angiograms, and optical coherence tomography, after treatment with intravitreal bevacizumab, which shows good response.

The cohort comprised 41 patients of which 21 were female and 20 male. The mean age of patients was 41 ± 16 years with a mean followup of 4 ± 1 months. Patients were divided into four groups: idiopathic intermediate uveitis (10 patients) (Figure 1); Behcet's disease (9 patients); idiopathic panuveitis (10 patients); and presumed ocular tuberculosis uveitis group (12 patients) (Figure 2).

The mean LogMAR visual acuity for the study cohort improved from a baseline value of 0.8 ± 0.8 to 0.4 ± 0.5 at 1 month and 0.3 ± 0.5 at 3 months. The improvement in visual acuity at 3 months was statistically significant (*P* < 0.002) (Table 1). There was a continuous increase in mean visual acuity over the duration of followup in each group (Table 1). The baseline macular thickness for the study cohort was 430 ± 132 *μ*m. Following intravitreal bevacizumab, the macular thickness improved to 286 ± 93 *μ*m at 1 month and to 265 ± 88 *μ*m at 3 months. The improvement in macular thickness at 3 months was statistically significant (*P* < 0.001) (Table 1).

The change in visual acuity and macular thickness for each group is presented in Table 1. All groups had an increase in mean visual acuity after intravitreal bevacizumab (Table 1). The greatest reduction in macular thickness occurred at 1 month in Behcet's disease group, but the edema reappeared by 3 months (Table 1). All other groups had a continuous reduction in macular thickness at 3 months (Table 1). The greatest reduction in macular thickness from baseline to 3 months occurred in the idiopathic intermediate uveitis group (Table 1).

Thirteen (32%) out of 41 patients received more than one intravitreal bevacizumab injection. Eight of these patients had uncontrolled intraocular inflammation and 5 (15%) of 33 patients (*P* < 0.001) had well-controlled intraocular inflammation.

No systemic or ocular complications were noted following intravitreal bevacizumab. A transient rise in intraocular pressure following intravitreal bevacizumab was observed in 14 (34%) patients.

## 4. Discussion

Uveitis is an important cause of ocular morbidity, as it can cause progressive, relentless destruction of visually important structures such as the macula. Immune-mediated inflammation of the uvea afflicts 1.15 per 1,000 individuals in the western hemisphere [37]. Chronic UME is frequently seen in patients with chronic uveitis. The therapeutic strategy for immune-mediated uveitis is evolving as new therapeutic modalities emerge. Immune-mediated insults initiate a chain of events at the cellular and molecular levels leading to an upregulation of several cytokines such as VEGF which is upregulated in patients with uveitis [5–8, 10].

Currently, there is no standard treatment for managing UME associated with chronic uveitis. Currently available treatment consists of topical nonsteroidal anti-inflammatory, oral, periocular, and intraocular injections of corticosteroids, as well as oral carbonic anhydrase inhibitors, systemic somatostatin analogs, and recently interferon alpha, mycophenolate mofetil, and VEGF inhibitors [11–20].

The outcomes of the current study indicate that intravitreal bevacizumab is effective, tolerable, and safe for the management of UME associated with uveitis. For example, there was a significant reduction in UME indicated by the decrease in macular thickness. Additionally, there was a concomitant improvement in visual acuity in patients suffering from idiopathic intermediate uveitis, panuveitis, Behcet's disease, and presumed ocular tuberculosis. These outcomes indicate that anti-VEGF treatment, which has no immunosuppressive effects may serve as a safe treatment for UME in patients with infectious uveitis. Our results concur with several reports that have described an improvement in macular edema and regression of ocular neovascularization following intravitreal bevacizumab for uveitis [7, 10, 29–31, 33]. The improvement of macular edema after intravitreal bevacizumab was transient and short-lived in several studies [30, 31, 38]. In this study, we found that adequate control of intraocular inflammation is associated with reduction in the number of intravitreal bevacizumab reinjection. Uncontrolled intraocular inflammation may lead to recurrence of UME which would warrant repeat injections of bevacizumab. We found that intravitreal bevacizumab with the control of inflammation affords long-term remission of UME. For example, only 5 out of 33 patients with controlled intraocular inflammation required more than one injection of intravitreal bevacizumab in comparison to 8 patients with uncontrolled active intraocular inflammation who received more than one injection (*P* < 0.001). Repeat injections were indicated in patients with active uveitis. We believe that bevacizumab is an important adjuvant treatment to appropriate therapies for the management of UME associated with infectious or noninfectious uveitis due to the lack of an immunosuppressive effect and the safety and efficacy.

Some limitations of this study include the retrospective review and short follow-up period. However, consecutive patients irrespective of outcome were selected over the time period of this study to mitigate some of the drawbacks.

In conclusion, cases with well-controlled intraocular inflammation that receive adjunct intravitreal bevacizumab result in long-term remission of UME. In cases of UME associated with infectious uveitis, the lack of immunosuppression from intravitreal bevacizumab treatment will not interfere with the immune response. Longer-term prospective studies are required to confirm the observation in this study.

## Figures and Tables

**Table 1:** Demographics, visual acuity, and macular thickness of patients with uveitic cystoid macular edema treated with intravitreal bevacizumab.

	IIU	BD	IPU	POTBU	*P* value
Number of patients	10	9	10	12	
Mean age	44 ± 16	34 ± 7	28 ± 13	43 ± 17	
Mean followup	4 ± 1	4 ± 1	4 ± 1	3.9 ± 2	
Mean number of Avastin injections	1.2 ± 0.4	1.7 ± 0.7	1.6 ± 0.7	1.6 ± 0.5	
Mean initial VA	0.5 ± 0.8	0.8 ± 0.8	0.8 ± 0.8	0.8 ± 0.5	
Mean 1-month VA	0.3 ± 0.4	0.4 ± 0.8	0.5 ± 0.8	0.5 ± 0.8	
Mean 3-month VA	0.2 ± 0.4	0.2 ± 0.5	0.3 ± 0.5	0.4 ± 0.5	<0.002
Mean initial OCT thickness (*μ*m)	437 ± 121	433 ± 179	342 ± 83	404 ± 134	
Mean OCT thickness (1 month) (*μ*m)	314 ± 120	259 ± 102	270 ± 45	296 ± 94	
Mean OCT thickness (3 months) (*μ*m)	246 ± 80	284 ± 106	239 ± 49	281 ± 110	<0.001

*P* value (ANOVA) was assessed for the mean OCT retinal thickness and the mean LogMAR change in visual acuity form baseline.

IIU: idiopathic intermediate uveitis, BD: Behcet's disease, IPU: idiopathic panuveitis, POTBU: presumed ocular tuberculosis uveitis, VA: visual acuity, and OCT: optical coherence tomography.
